# Non-Polar Natural Products from *Bromelia laciniosa*, *Neoglaziovia variegata* and *Encholirium spectabile* (Bromeliaceae)

**DOI:** 10.3390/molecules22091478

**Published:** 2017-09-06

**Authors:** Ole Johan Juvik, Bjarte Holmelid, George W. Francis, Heidi Lie Andersen, Ana Paula de Oliveira, Raimundo Gonçalves de Oliveira Júnior, Jackson Roberto Guedes da Silva Almeida, Torgils Fossen

**Affiliations:** 1 Department of Chemistry and Centre for Pharmacy, University of Bergen, Allégaten 41, 5007 Bergen, Norway; Ole-Johan.Juvik@uib.no (O.J.J.); Bjarte.Holmelid@uib.no (B.H.); George.Francis@kj.uib.no (G.W.F.); 2 Arboretum and Botanical Gardens, University of Bergen, Allégaten 41, 5007 Bergen, Norway; Heidi.Andersen@uib.no; 3 Centre for Studies and Research of Medicinal Plants, Federal University of Vale do São Francisco, 56.304-205 Petrolina, Pernambuco, Brazil; ana_tecquimica@yahoo.com.br (A.P.d.O.); oliveira.farma.junior@gmail.com (R.G.d.O.J.), jackson.guedes@univasf.edu.br (J.R.G.d.S.A.)

**Keywords:** *Bromelia laciniosa*, *Neoglaziovia variegata*, *Encholirium spectabile*, nonpolar natural products, Hyphenated GC-HRMS, 2D NMR plant metabolomics

## Abstract

Extensive regional droughts are already a major problem on all inhabited continents and severe regional droughts are expected to become an increasing and extended problem in the future. Consequently, extended use of available drought resistant food plants should be encouraged. *Bromelia laciniosa*, *Neoglaziovia variegata* and *Encholirium spectabile* are excellent candidates in that respect because they are established drought resistant edible plants from the semi-arid Caatinga region. From a food safety perspective, increased utilization of these plants would necessitate detailed knowledge about their chemical constituents. However, their chemical compositions have previously not been determined. For the first time, the non-polar constituents of *B. laciniosa*, *N. variegata* and *E. spectabile* have been identified. This is the first thorough report on natural products from *N. variegata*, *E. spectabile*, and *B. laciniosa*. Altogether, 20 non-polar natural products were characterized. The identifications were based on hyphenated gas chromatography-high resolution mass spectrometry (GC-HRMS) and supported by 1D and 2D Nuclear Magnetic Resonance (NMR) plant metabolomics.

## 1. Introduction

### 1.1. Bromeliaceae

**Figure 1:**
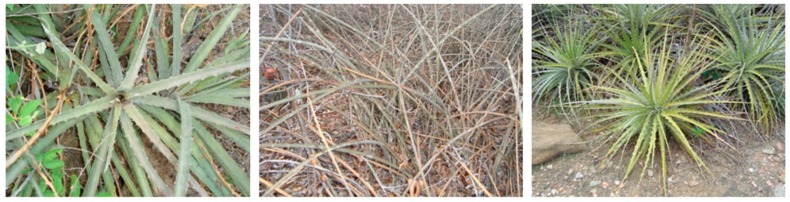
*Bromelia laciniosa* (**left**); *Neoglaziovia variegata* (**middle**); and *Encholirium spectabile* (**right**) grown in Petrolina, Pernambuco, Brazil. Photos: JRGS Almeida.

Several recent severe regional droughts have led to increased interest in exploiting drought resistant edible plants as human food sources and as forage for domesticated animals. Several drought-tolerant plants already utilized for such purposes belong to the Bromeliaceae and display features that make them especially capable of retaining water; the leaves are shaped adaxially concave to channel rainwater down to the overlapping, rosulate base for storage in a central cavity. The surface of the leaves bears absorptive, scale-like trichomes that take up water and nutrients. Bromeliaceae also have a Crassulacean acid *metabolism* (CAM) photosynthesis, where the stomata remain shut during daytime to avoid evaporation. The Bromeliaceae is a large family of flowering plants within the Monocots, which contains 58 genera and approximately 3200 species. Except for one western African species, all Bromeliaceae are endemic to the American tropics. The family is currently divided into eight subfamilies [1], and the two species, *Bromelia laciniosa* Mart. ex Schult. & Schult. f. (Figure 1) and *Neoglaziovia variegata* (Arruda) Mez. (Figure 1), which belong to the subfamily Bromelioideae, and *Encholirium spectabile* Mart. ex Schult. & Schult. f (Figure 1),which belongs to the Pitcairnioideae subfamily. Bromeliads are used for alimentation (e.g., fruit production of *Ananas comosus* (L.) Merr., as fiber plants, and cultivated for ornamental and medicinal purposes.

### 1.2. B. laciniosa

*B. laciniosa* (Portuguese: macambira de porco) is native to Brazil and Argentina. Leaves from *B. laciniosa* are rich in proteins (4.9%), starch (2.8%) and calcium (1.1%) [2], and are therefore used in alimentation of both humans and domestic animals in northeastern Brazil [3,4]. Farmers in this region use the leaves as supplementary fodder for livestock [3,5,6]. The leaves are dried, powdered and mixed into the local cuisine [7]. A type of bread can be made from masses extracted from the base of the leaves [3]. Flowers, fruit and leaves from *B. laciniosa* are used in the treatment of infantile colic, diarrhea, fever, jaundice, hepatitis, and dandruff [8]. An aqueous root extract may be drunk as a treatment against intestinal diseases and hepatitis, and as a diuretic [7]. Only limited information is available which could rationalize these medicinal applications. Gum from *B. laciniosa* has been shown to contain galactose, arabinose and xylose, and an acidic oligosaccharide composed of xylose and galacturonic acid [9]. Quercetin 3,3′,4′-trimethyl ether is the only natural product isolated and characterized from *B. laciniosa* [10].

### 1.3. N. variegata

*N. variegata* (Portuguese: caroá) is one of only three species in the genus *Neoglaziovia*, all of which are endemic to northeastern Brazil. At the beginning of the rainy season, *N. variegata* produces edible fleshy fruits [11]. The plant was first described by the Brazilian Manuel Arruda da Câmara (1752–1810), while the genus is named after the French botanist Auguste Francois Marie Glaziou (1828–1906). *N. variegata* is used as a fiber plant by rural communities in the Caatinga region where a variety of products are made from the white, soft and flexible fibers [12,13,14,15]. Ethanol extracts of *N. variegata* have been reported to be of low toxicity [16] , and to exhibit antinociceptive effect in experimental models in mice [16], photoprotective potential, antioxidant effect [13,16], gastroprotective effects in a mice model of gastric ulcer [17] and antibacterial effect against both Gram-positive [13] and Gram-negative bacteria [13,18]. There is no report of natural products characterized from *N. variegata*.

### 1.4. E. spectabile

*E. spectabile* (Portuguese: macambira de flexa or macambira de pedra) is one of twenty five species in this genus, which is endemic to Brazil. This species is used as a supplementary food supply in famine emergencies by rural communities in the semi-arid Caatinga region [19]. The edible part of *E. spectabile* is the leaf base, which is rich in carbohydrates (28.7%), and contains some proteins (0.7%) and lipids (0.8%) [19]. Flour made from the dried leaves is used to prepare a somewhat bitter tasting couscous. The nutritional value of *E. spectabile* (124.6 kcal/100 g) is in the same range as commercial grains such as rice (*Oryza sativa* L.) with 130 kcal/100 g [19]. Extracts have exhibited no signs of toxicity towards mice [20]; have been reported to exhibit antioxidant [21,22], photoprotective [22], and anti-nociceptive activity in mice models [20]; gastroprotective activity in a mice model of gastric ulcer [23]; and antibacterial activity towards Gram-negative [18,21] and Gram-positive bacteria [21]. No natural products have been characterized from this species.

Very little authoritative information is available about natural products from *Bromelia laciniosa*, and there is absolutely no previous information about *Encholirium spectabile* and *Neoglaziovia variegata*. In this paper, we report for the first time on the natural products characterized from hexane extracts of *Bromelia laciniosa*, *Encholirium spectabile* and *Neoglaziovia variegata*.

## 2. Results and Discussion

**Figure 2:**
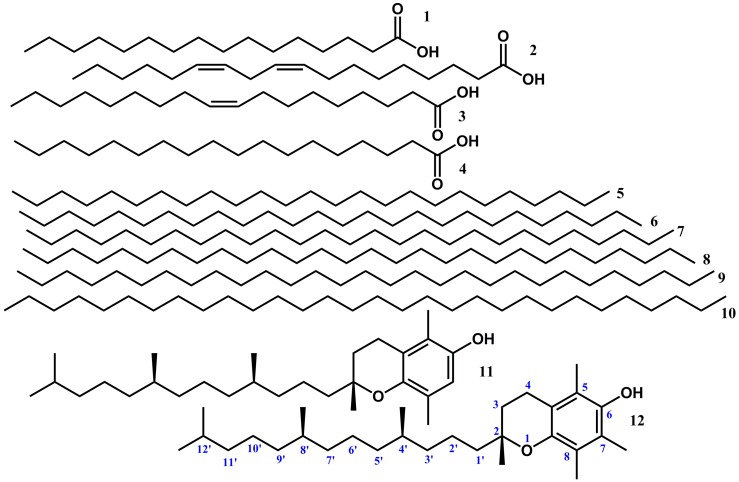
Structures of non-polar compounds identified from the leaves of *B. laciniosa*, *N. variegata* and *E. spectabile.* **1** *n*-Hexadecanoic acid (Palmitic acid); **2** 9*Z*,12*Z*-Octadecadienoic acid; **3** 9*Z*-Octadecenoic acid (Oleic acid); **4** Octadecanoic acid (Stearic acid); **5** *n*-Pentacosane; **6** *n*-Hexacosane; **7** *n*-Heptacosane; **8** *n*-Octacosane; **9** *n*-Nonacosane; **10** *n*-Triacontane; **11** β-Tocopherol; and **12** α-Tocopherol (Vitamin E).

**Figure 3:**
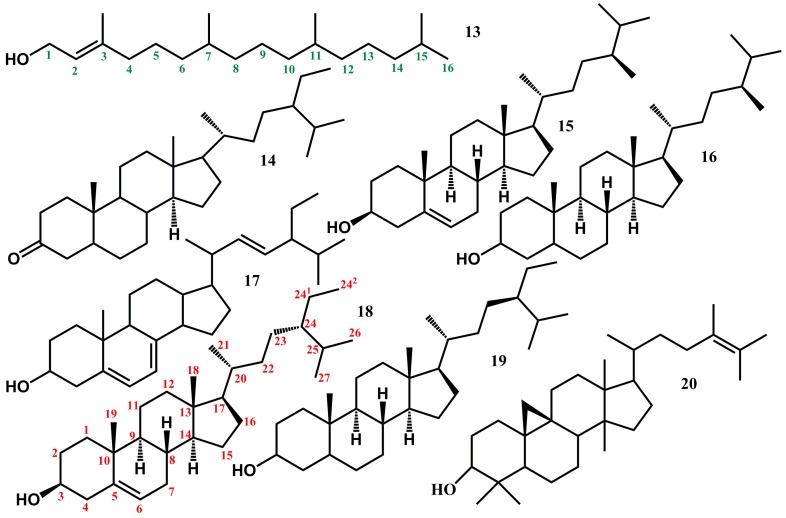
Structures of non-polar compounds identified from the leaves of *B. laciniosa*, *N. variegata* and *E. spectabile.* **13** Phytol; **14** Stigmastan-3-one; **15** Campesterol; **16** Ergostanol; **17** Stigmasta-4,22-dien-3-β-ol; **18** β-Sitosterol; **19** Stigmastanol; and **20** 24-Methyl-3-β -9,19-cyclolanost-24-en-3-ol.

**Figure 4:**
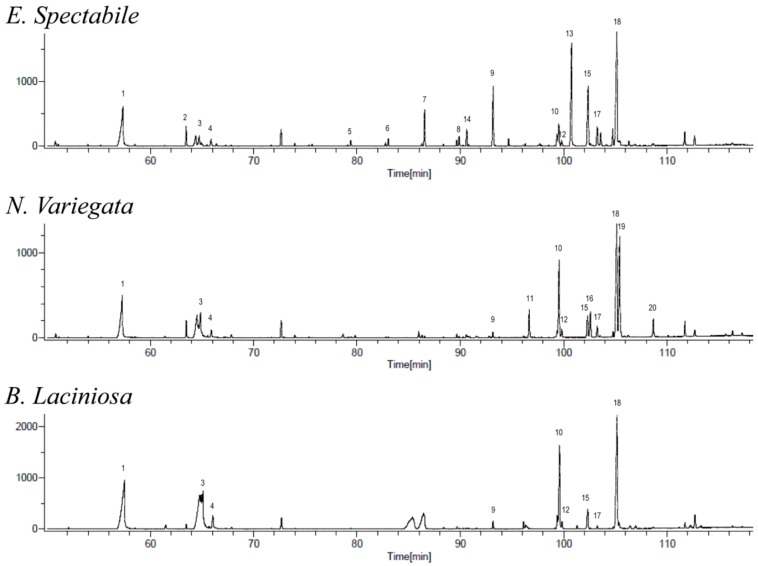
Comparison of TIC for *B. laciniosa*, *N. variegata* and *E. spectabile*.

As part of our ongoing work on the characterization of natural products from food and medicinal plants aimed at rationalizing the molecular basis of their applications, the constituents of non-polar extracts of *B. laciniosa*, *N. variegata* and *E. spectabile* have been characterized. All identifications were based on hyphenated GC-HRMS. Altogether, 20 compounds were for the first time identified in the chromatograms of the hydrophobic crude extracts of *B. laciniosa*, *N. variegata* and *E. spectabile* (Table 1 and Figure 2, Figure 3 and Figure 4). Compounds of each class are treated in separate paragraphs below.

### 2.1. Fatty Acids and Their Derivatives

Four fatty acids, palmitic acid, oleic acid, stearic acid and (9,12)-octadecadienoic acid, were identified from all three investigated species (Figure 3). These fatty acids are common phytochemical constituents from several plant species with significant nutritional value including wheat [24].

### 2.2. Very Long-Chain Alkanes (VLCA)

Very long-chain alkanes (VLCA) are considered to be of interest from a dietary point of view because their long-chain alcohol metabolites may contribute to the cholesterol-lowering effect associated with the intake of plant waxes [25]. The efficiency with which alkanes might contribute to a cholesterol-lowering effect of waxes is regulated by limited absorption and the need for hydroxylation of these compounds. Conversion to long-chain alcohols in vivo is achieved by the action of different cytochrome P450 enzymes, which hydroxylate alkanes at several positions [26]. Altogether, six very long-chain *n*-alkanes were identified (Figure 3). The identifications were initially based on the fragmentation pattern observed in the mass spectra. To further support these identifications, a standard solution containing alkanes with chain lengths varying from C_9_–C_40_ was co-chromatographed with the samples. The results confirmed the presence and identities of several long-chain alkanes. The six *n*-alkanes identified in this work have a chain length ranging from C_25_ to C_30_. *E. spectabile* differs from the two other species since the crude extract from this species contain all six *n*-alkanes from *n*-pentacosane (C_25_) to *n*- triacontane (C_30_). Meanwhile, only the two longest *n*-alkanes, *n*-nonacosane (C_29_) and *n*-triacontane (C_30_), were identified from the crude extracts from *B. laciniosa* and *N. variegata*.

The quantitative amount of the two *n*-alkanes identified from all of the species seems to be largest in the crude extract from *E. spectabile* while the amount in the crude extracts from *B. laciniosa* and *N. variegata* was much lower and at a comparable level. Seven alkanes with considerably shorter chain lengths, ranging C_12_–C_18_, have previously been identified from Bromeliaceae spp. [27]. Of particular importance may be that the chain lengths of the alkanes identified in *B. laciniosa*, *N. variegata* and *E. spectabile* were considerably longer than for those that have previously been reported to occur in Bromeliaceae species.

### 2.3. Vitamins

α-Tocopherol was present in all three investigated species (Figure 2). The discovery of the presence of an active form of vitamin E in the crude extracts from *B. laciniosa* and *E. spectabile* underlines the nutritional value of these plants as food for both humans and animals. Previously, α-tocopherol has not been identified from any plant source belonging to genus *Bromelia*. However, the vitamin has been found in the distant relative *A. erectifolius* belonging to genus *Ananas* in Bromeliaceae [28]. Moreover, another vitamin E, namely β-tocopherol (Figure 2), was identified in the crude extract of *N. variegata*.

### 2.4. Other Compounds

The relatively common natural product phytol (Figure 3) was identified in the crude extract from *E. spectabile*. According to Vetter et al. (2012), the presence of phytol in human food is mainly restricted to spinach, beans, raw vegetables, and asparagus [29,30]. It may be mentioned that intake of food plants containing free phytol should be restricted for individuals suffering from Refsum’s disease [30].

### 2.5. Triterpenoids and Derivatives Therefrom

Altogether, seven triterpenoids were identified from the investigated Bromeliaceae species (Figure 4). While all seven were detected in *N. variegata,* only three (Compounds **15**, **17** and **18**) were identified in *B. laciniosa* and *E. spectabile.* With twice as many identified triterpenoids, *N. variegata* differs markedly from the two other species (Table 1). Five of the seven triterpenoids have previously been identified from other species of the family Bromeliaceae (Compounds **15**–**19**). The two triterpenoids stigmastan-3-one (Compound **14**) and 24-methyl-β-9,19-cyclolanost-24-en-3-ol (Compound **20**) are identified from species of the family Bromeliaceae for the first time. 24-Methyl-β-9,19-cyclolanost-24-en-3-ol (Compound **20**) is also known as 24-methyl-cycloartenol. Campesterol (Compound **15**), ergostanol (Compound **16**), stigmasta-4,22-dien-3-β-ol (Compound **17**), β-sitosterol (Compound **18**) and stigmastanol (Compound **19**) have all been detected previously from species of the family Bromeliaceae. Although stigmastan-3-one (Compound **14**) has not been identified from Bromeliaceae previously the unsaturated form stigmast-4-en-3-one has previously been identified from *Ananas erectofolius* [28]. Campesterol (Compound **15**) is a common phytosterol found in many edible plants. It is therefore unsurprising that campesterol has been identified from six Bromeliaceae species. The species are *A. comosus* [31], *A. erectofolius* [28], *Tillandsia fasciculata* [32], *Tillandsia pohliana* (*T. pohliana* has been examined by Caiado and co-workers in an unpublished work according to Manetti et al. [33]), *Tillandsia streptocarpa* [34] and *T. usneoides* [35]. Ergostanol (Compound **16**) has previously been identified from the two species *A. comosus* [31] and *A. erectofolius* [28] from the family Bromeliaceae. Stigmasta-4,22-dien-3-β-ol (Compound **17**) is an unsaturated derivative of stigmastanol, which is known to inhibit absorption of cholesterol from the diet. Stigmasta-4,22-dien-3-β-ol has previously been identified from the three species *T. fasciculata* [32], *T. streptocarpa* [34] and *T. usneoides* [35]. All three species belongs to *Tillandsia,* a genus of the Bromeliaceae family. β-Sitosterol (Compound **18**) is the major compound from the non-polar (hydrophobic) extracts in all three investigated species. β-Sitosterol (Compound **18**) is a phytosterol with wide distribution throughout the plant kingdom including several plants used for human nutrition. Several dietary plant sterols including β-sitosterol exhibit significant cholesterol-lowering effects [36,37,38]. In the Bromeliaceae family β-sitosterol has been identified from the eight species: *A. comosus* [31,39] commonly known as pineapple, *A. erectofolius* [28], *Hechtia rosea* [40], *H. scariosa* [40], *T. fasciculata* [32], *T. pohliana* [33], *T. streptocarpa* [34], *T. usneoides* [35,41]. Stigmastanol (Compound **19**) is previously known from two species of the Bromeliaceae family, namely *A. comosus* [31] and *A. erectofolius* [28]. Stigmastanol is also known as sitostanol and is a phytosterol commonly found in many edible plants. 9,19-Cyclolanost-24-en-3-ol-3-β (Compound **20**) is not previously identified from species of the Bromeliaceae family. However, similar cycloartanol triterpenoids such as cyclolaudenol [32] and 24-methylenecycloartanol [35,42] are commonly found in Bromeliaceae species [33]. Identification of phytosterols with documented potential beneficial health effects, such as campesterol and β-sitosterol, from the leaves of *B. laciniosa* and *E. spectabile* strengthens the nutritional value of these plants.

### 2.6. NMR Plant Metabolomics

Even though GC-HRMS is an excellent method for characterizing mixtures of natural products, there are some limitations in connection with the application of this method. Some compounds may avoid detection because they are either not sufficiently volatile or insufficiently ionized. To further support the identifications of natural products achieved by GC-HRMS, we proceeded with a NMR metabolomics strategy by directly analyzing the dried extract of *N. variegata* (when dissolved in deuterated chloroform) on 600 MHz NMR equipped with a cryogenic probe, without any requirements for further workup of the sample. NMR plant metabolomics is, among others, a complementary strategy for identification of known plant metabolites of plant-derived extracts and allows for the detection of signals of all compounds present in the sample at sufficient quantities to be detected. Recent development in cryoprobe technology has made it possible to characterized complex natural products with concentrations as low as in the micromolar range. In current literature, NMR spectroscopy has been successfully applied in the evaluation of metabolites of plant extracts (NMR plant metabolomics) [43]. NMR metabolomics has gained importance because this strategy provides insight into complex systems of mixtures of natural products occurring at their natural relative abundance. NMR is able to provide a “holistic view” of the metabolites under certain conditions, and thus is advantageous for metabolomic studies [44]. Although most publications about NMR metabolomics, including plant metabolomics, only include application of 1D ^1^H NMR (reviewed by Kim et al. 2011 [44]), several applications of 2D NMR exist in plant metabolomics [45]. Recent development in cryoprobe technology has led to a four-fold increase of sensitivity and thus a 16-fold reduction of experiment time for 2D inverse experiments compared with those for similar NMR experiments recorded on analogous instruments equipped with conventional probes. This allows for applications of a broad selection of 2D NMR spectroscopic experiments, which are now accessible within an acceptable time scale of approximately 15–30 min per experiment.

**Figure 5:**
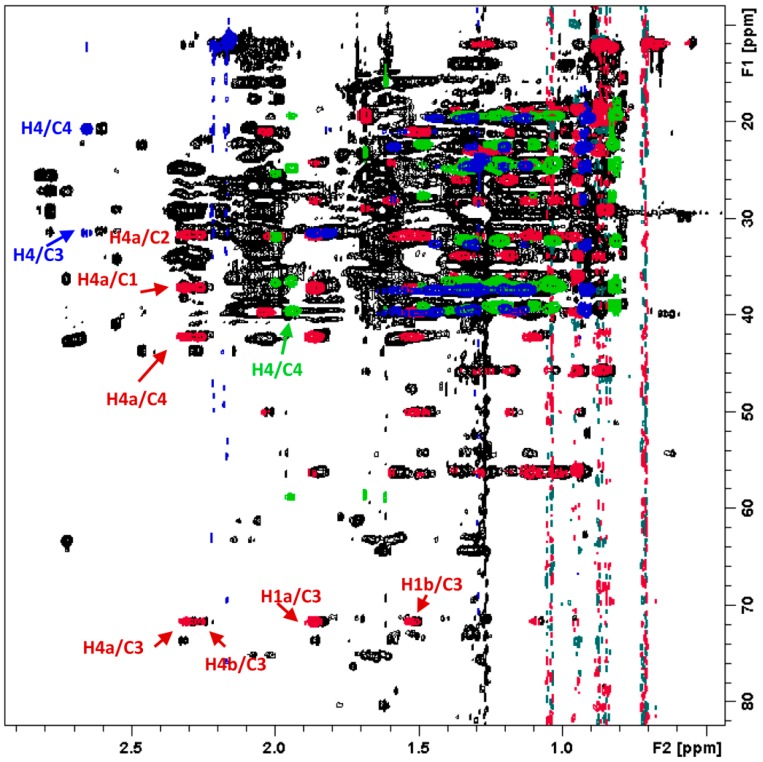
Expanded region of the superimposed 2D ^1^H-^13^C HSQC-TOCSY NMR spectra of *N. variegata* (black signals), β-sitosterol (red signals), phytol (green signals) and α-tocopherol (blue signals). Selected crosspeaks are assigned. Complete assignments are presented in Tables S1–S3. Notice that the characteristic signal patterns of the pure standard compounds matches the corresponding signals of the same compounds present as part of mixture comprising the extract of *N. variegata*.

To support the identifications achieved with GC-HRMS, 1D ^1^H (Figure S1) and the 2D NMR experiments 2D ^1^H-^13^C Heteronuclear Single Quantum Coherence (HSQC), 2D ^1^H-^13^C Heteronuclear Multiple Bond Correlation (HMBC) (Figure S2), 2D ^1^H-^13^C Heteronuclear Single Quantum Coherence-Total Correlation Spectroscopy (HSQC-TOCSY), 2D ^1^H-^13^C Heteronuclear 2 Bond Correlation (H2BC), 2D ^1^H-^1^H Correlation Spectroscopy (COSY) and 2D ^1^H-^1^H Rotating frame Overhauser enhancement spectroscopy (ROESY) of the dried hexane extract of *B. laciniosa* dissolved in deuterated chloroform were recorded. The same NMR experiments were also recorded on samples of pure β-sitosterol, stigmasterol, α-tocopherol and phytol. The combined information from the 2D ^1^H-^13^C edited HSQC, HSQC-TOCSY, HMBC and H2BC were particularly helpful for assignment of ^1^H and ^13^C signals of both reference compounds (Tables S1–S3) and the analogous signals belonging to the mixture containing these compounds comprising the extract sample. The overlaid 2D NMR spectra of extract of *N. variegata* and pure β-sitosterol, α-tocopherol and phytol confirmed the presence of these compounds in the plant extract and allowed for identifications of individual signals (Figure 5). The multidimensional NMR data provided supportive evidence for the presence of the above-mentioned compounds, as well as significant amounts of long-chain alkanes and fatty acids identified by hyphenated GC-HRMS in *N. variegata* (Table 1).

## 3. Materials and Methods

### 3.1. Plant Material

Leaves of *B. laciniosa*, *E. spectabile* and *N. variegata* were collected within the municipality borders of Petrolina, Pernambuco, Brazil, in January 2013. Voucher specimens were deposited in the Herbarium Vale do São Francisco (HVASF) of the Federal University of Vale do São Francisco. The site for collecting the leaves of *B. laciniosa* was at 08°59′16.90′′ S and 40°35′20.60′′ W and the voucher specimen is No. 6442. The leaves of *E. spectabile* were collected at the coordinates 09°07′54.30′′ S and 40°26′21.00′′ W and the voucher specimen is No. 6443. *N. variegata* leaves were collected at the coordinates 08°59′16.90′′ S and 40°35′20.60′′ W and the voucher specimen is No. 6441. Identification of the collected plant species was done by the botanist André Paviotti Fontana from Centro de Recuperação de Áreas Degradadas da Caatinga (CRAD). Prior to shipment to Norway the leaves were dried in an oven with air circulation at a temperature of 50 °C for seven days. After drying, the plant materials were powdered in a mill.

### 3.2. Extraction and Concentration

Dried and pulverized leaves of *B. laciniosa* (100.86 g), *E. spectabile* (100.22 g) and *N. variegata* (100.49 g) were separately macerated in 800 mL of hexane for 89 h at room temperature. After extraction, solutions were filtered through glass wool before being concentrated under reduced pressure on a rotary evaporator. The volumes were reduced to 140 mL (*B. laciniosa*), 75 mL (*E. spectabile*) and 145 mL (*N. variegata*), respectively. Before examination by GC-FID and GC-HRMS the concentrated samples were filtered through a 0.45 µm Micropore Membrane Filter.

### 3.3. GC-FID

To optimize conditions for the GC-MS analysis, GC-FID was performed. These investigations of the composition and concentration of the extracts were performed on a Gas Chromatograph (GC) with a Flame Ionization Detector (FID). A Trace GC Ultra instrument (Thermo Electron Corporation S.p.A., Milan, Italy) fitted with an Ultra 1 column (crosslinked methyl siloxane, ID = 0.200 mm, L = 25 m, film thickness = 0.33 μm) (Santa Clara, CA, USA). Samples were dissolved in hexane and splitless mode was used for injection. The applied temperature gradient (initial temperature = 50 °C, holding for 2.5 min, then heating at 20 °C/min to 100 °C, holding for 10 min, finally heating at 2 °C/min to 300 °C applying a 15 min holding time provided both good separation of the compounds and the necessary information about the concentration of the extracts. Helium was used as carrier gas with a flow rate of 0.7 mL/min. An injector temperature of 260 °C was used.

### 3.4. GC-MS (TOF)

All samples were analyzed on an AccuTOF T100GC mass spectrometer from JEOL Ltd. (Tokyo, Japan) interfaced with an Agilent 6890 N gas chromatograph (Santa Clara, CA, USA). Samples dissolved in hexane were injected on a VF-50 MS GC column from Varian Inc. (Palo Alto, CA, USA), (silica column (5% phenyl)-metylpolysiloxane, ID = 0.200 mm, L = 25 m, film thickness = 0.33 μm) using splitless injection at 250 °C (injector temperature). Helium (5.0) was used as carrier gas at a constant gas flow rate of 0.7 mL/min (ʋ = 33.0 cm/s), and the following GC temperature program was applied; initial temperature = 40 °C, holding for 2.5 min, then heating at 20 °C/min to 100 °C, holding for 10 min, finally heating at 2 °C/min to 300 °C applying a 15 min holding time. The GC-MS interface was heated to 260 °C introducing the column flow into the electron ionization source four minutes after injection. The ion source operated at 260 °C generating positive ions at an ionization potential and ionization current of 70 eV and 300 μA, respectively. Settings for the time of flight mass analyser were optimized for ions in the mass range 40–800 amu, acquiring mass spectra after the following acquisition settings; spectral recording interval = 0.3 s, wait time = 0.005 s, spectra accumulation time = 0.295 s and data sampling interval = 1 ns. A total ion chromatogram (TIC) was acquired during the whole GC run and mass spectra were generated and transformed to centroided spectra using baseline correction and smoothing using a weighted moving average. All mass spectra were calibrated against one of several polysiloxane background ions (*m*/*z* = 147.03290, 207.03290, 281.05169 or 355.07048) originating from the column and acquired during the same set of experiments. The relative quantities of each compound were calculated based on the peak heights in the total ion chromatogram. Mass spectral fragmentation patterns of individual compounds was compared with that of analogous standard compounds in NIST standard reference Mass Spectral library. Absolute configurations are based on mass spectral matches against known library mass spectra (NIST07), when applicable. Individual mass spectra are shown in Figures S3–S16.

### 3.5. NMR Spectroscopy

NMR spectroscopy was performed on samples of 19.1 mg dried heptane extract of *B. laciniosa* and on 164.9 mg dried hexane extract of *N. variegata*. In addition to pure samples of 20.0 mg of β-sitosterol, stigmasterol, 25 volume % of phytol and 76.2 mg of α-tocopherol, each individual sample was dissolved in, or mixed with, 0.75 mL chloroform-D. The 1D ^1^H and the 2D ^1^H-^1^H COSY, 2D ^1^H-^1^H ROESY, the 2D ^1^H-^13^C HSQC, the 2D ^1^H-^13^C Edited HSQC, the 2D ^1^H-^13^C HSQCTOCSY, the 2D ^1^H-^13^C HMBC and the 2D ^1^H-^13^C H2BC NMR experiments were recorded on a Bruker Avance 600 MHz spectrometer (Bruker BioSpin AG, canton of Zürich, Switzerland) equipped with a ^1^H-^13^C-^15^N triple resonance cryoprobe at 298 K. All 2D NMR experiments were recorded without spinning.

## 4. Conclusions

Using a combination of hyphenated GC-HRMS and NMR plant metabolomics, 20 natural products have been identified in the drought-resistant plants *B. laciniosa*, *N. variegata* and *E. spectabile* for the first time. A total of 13 natural products including six triterpenoids were identified from *N. variegata*. From the edible leaves of *B. laciniosa* and *E. spectabile,* 9 and 16 natural products were identified, respectively. The presence of significant amounts of vitamin E in leaves of *B. laciniosa* and *E. spectabile*, as well as nutrients such as fatty acids, and phytosterols with well documented potential beneficial health effects, as well as the absence of compounds with significant toxicity, underlines the nutritional values of the plants as food sources for humans and livestock.

## Figures and Tables

**Table 1:** Compounds identified from hexane extracts of leaves of *B. laciniosa*, *N. variegata* and *E. spectabile*.

Nr.	Compounds According to Group	MF	Exact Mass *		Retention Time (min)			Content (%)		
			Observed	Calculated	*B. L.*	*N. V.*	*E.S.*	*B.L.*	*N.V.*	*E.S.*
	**Fatty acids and their derivatives**									
1	*n*-Hexadecanoic acid (Palmitic acid)	C_16_H_32_O_2_	256.24110	256.24023	57.52	57.30	57.38	11.3	7.4	6.1
2	Octadeca-(9,12)dienoic acid	C_18_H_32_O_2_	280.24398	280.24023			64.38			3.1
3	(9*Z*)-Octadec-9-enoic acid (Oleic acid)	C_18_H_34_O_2_ C_18_H_32_O ^a^	n.a. 264.2434	264.24532	65.10	64.86	64.75	8.8	4.5	1.7
4	Octadecanoic acid (Stearic acid)	C_18_H_36_O_2_	284.27187	284.27153	66.05	65.88	65.88	3.2	1.4	1.1
	**Alkanes**									
5	*n*-Pentacosane	C_25_H_52_					79.41			0.8
6	*n*-Hexacosane	C_26_H_54_					83.03			1.1
7	*n*-Heptacosane	C_27_H_56_					86.54			5.7
8	*n*-Octacosane	C_28_H_58_					89.90			1.4
9	*n*-Nonacosane	C_29_H_60_			93.16	93.14	93.18	2.0	1.0	9.3
10	*n*-Triacontane	C_30_H_62_			99.36	99.34	99.35	18.9	13.6	3.5
	**Vitamins**									
11	β-Tocopherol	C_28_H_48_O_2_	416.36392	416.36543		96.67			4.9	
12	α-Tocopherol	C_29_H_50_O_2_	430.38089	430.38108	99.60	99.56	99.52	1.8	1.5	0.9
	**Phytol**									
13	(2*E*,7*R*,11*R*)-3,7,11,15-Tetramethyl-2-hexadecen-1-ol (Phytol)	C_20_H_40_O_1_	296.30982	296.30791			100.76			16.1
	**Triterpenoids and derivatives**									
14	Stigmastan-3-one	C_29_H_50_O_1_	414.38688	414.38616			90.62			2.6
15	Campesterol ((3β,24*R*)-Ergost-5-en-3-ol)	C_27_H_44_O_2_	400.33890	400.33413	102.33	102.33	102.37	4.5	3.9	9.4
16	Ergostanol	C_28_H_50_O_1_	402.38723	402.38616		102.60			4.5	
17	Stigmasta-4,22-dien-3-β-ol	C_29_H_48_O_1_	412.37123	412.37051	103.24	103.24	103.25	0.7	2.0	3.1
18	β-Sitosterol	C_29_H_50_O_1_	414.38655	414.38616	105.18	105.14	105.16	25.8	19.8	17.8
19	Stigmastanol	C_29_H_52_O_1_	416.40276	416.40181		105.42			17.6	
20	24-Methyl-3-β-9,19-cyclolanost-24-en-3-ol	C_31_H_52_O_1_	440.40234	440.40181		108.67			3.2	
	Unidentified							23.0	14.7	16.3

Abbreviations: *B. L.* = *B. laciniosa*; *N. V.* = *N. variegata*; *E. S.* = *E. spectabile*; n.d. = not detected; MF = Molecular Formula. ^a^ Only the pseudomolecular ion [M − H_2_O]^+^ observed for this compound. * All exact masses are calculated for lowest monoisotopic mass.

## Supplementary Materials

Supplementary materials are available online.
