# Supplementary material for: Non-Polar Natural Products from Bromelia laciniosa, Neoglaziovia variegata and Encholirium spectabile (Bromeliaceae)
Source: Molecules. 2017 Sep 6;22(9):1478. doi: 10.3390/molecules22091478 (PMC6151397; doi:10.3390/molecules22091478)
Supplement: Supplementary file 1 [file molecules-22-01478-s001.pdf]

Supplementary data

Non-polar natural products from *Bromelia laciniosa, Neoglaziovia variegata* and *Encholirium spectabile* (Bromeliaceae)

Ole Johan Juvik[a], Bjarte Holmelid[a], George W. Francis[a], Heidi Lie Andersen[b], Ana Paula de Oliveira[c], Raimundo Gonçalves de Oliveira Júnior[c], Jackson Roberto Guedes da Silva Almeida[c], Torgils Fossen[a]*

Table S1. ^{1}H and ^{13}C NMR data of β-sitosterol in $CDCl_3$ at 298K.

	^{1}H ppm	^{13}C ppm
1a	1.83	37.3
1b	1.06	
2a	1.81	31.7
2b	1.48	
3	3.50	71.8
4a	2.26	42.3
4b	2.21	
5		140.8
6	5.33	121.7
7a	1.96	31.8
7b	1.50	
8	1.43	32.0
9	0.91	50.1
10		36.5
11	1.46	21.2
12a	1.99	39.9
12b	1.13	
13		42.2
14	0.97	56.7
15a	1.56	24.3
15b	1.05	
16a	1.82	28.3
16b	1.24	
17	1.08	56.0
18	0.66	11.9
19	0.99	19.3
20	1.33	36.1
21	0.90	18.7
22a	1.48	21.2
22b	1.44	
23	1.14	26.0
24	0.91	45.8
24^{1}a	1.25	23.2
24^{1}b	1.20	
24^{2}	0.83	12.0
25	1.64	29.2
26	0.79	18.0
27	0.81	19.7

Table S2. ^{1}H and ^{13}C NMR data of α-tocopherol in $CDCl_3$ at 298K.

	^{1}H ppm	^{13}C ppm
2		74.5
3a	1.82	31.5
3b	1.77	
4	2.61	20.8
5		118.4
6		144.5
7		121.0
8		122.6
9		145.5
10		117.3
1a’	1.58	39.8
1b’	1.51	
2a’	1.46	21.0
2b’	1.39	
3’	1.79	37.5
4’	1.41	32.7
5a’	1.26	37.4
5b’	1.09	
6a’	1.26	37.4
6b’	1.09	
7a’	1.26	37.4
7b’	1.09	
8’	1.38	32.8
9a’	1.26	37.4
9b’	1.09	
10a’	1.32	24.7
10b’	1.24	
11’	1.16	39.4
12’	1.54	27.9
2-Me	1.24	23.8
5-Me	2.12	11.2
6-OH	4.20	
7-Me	2.17	12.2
8-Me	2.12	11.8
4’-Me	0.87	19.7
8’-Me	0.86	19.8
12’-Me	0.88	22.6
12’-Me	0.88	22.5

Table S3. ^{1}H and ^{13}C NMR data of phytol in $CDCl_3$ at 298K.

	^{1}H ppm	^{13}C ppm
1	4.08	59.0
2	5.35	123.4
3		139.7
4	1.94	39.7
5	1.34	25.0
6a	1.22	36.5
6b	1.03	
7	1.34	32.4
8a	1.21	37.1
8b	1.03	
9	1.22	24.4
10a	1.21	37.1
10b	1.03	
11	1.34	32.4
12a	1.21	37.1
12b	1.03	
13	1.21	24.6
14	1.09	39.1
15	1.48	27.7
16	0.82	22.4
17	0.82	22.4
18	0.81*	19.6
19	0.80*	19.6
20	1.61	15.9

*assignment may be reversed

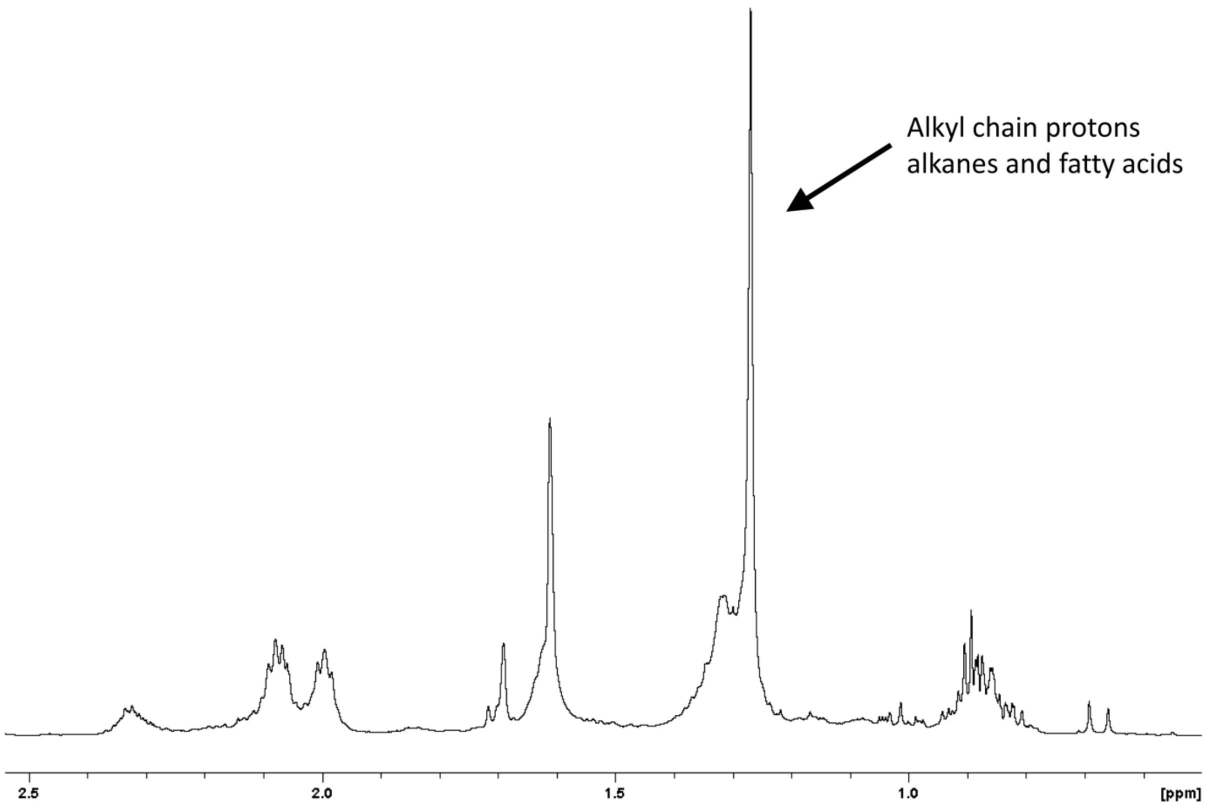

Figure S1A. Expanded region of the 1D ^{1}H NMR spectrum of hexane extract of *N. variegata*. The signal accounting for the majority of the alkyl protons of alkanes and fatty acids is highlighted.

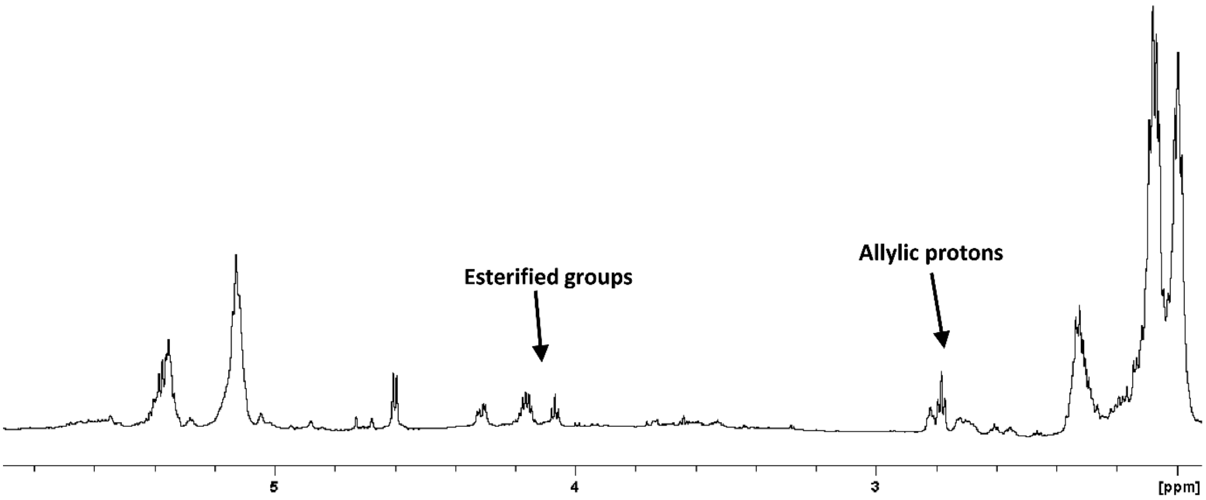

Figure S1B. Expanded region of the 1D ^{1}H NMR spectrum of hexane extract of *N. variegata* including the regions for signals belonging to esterified groups and allylic protons, respectively.

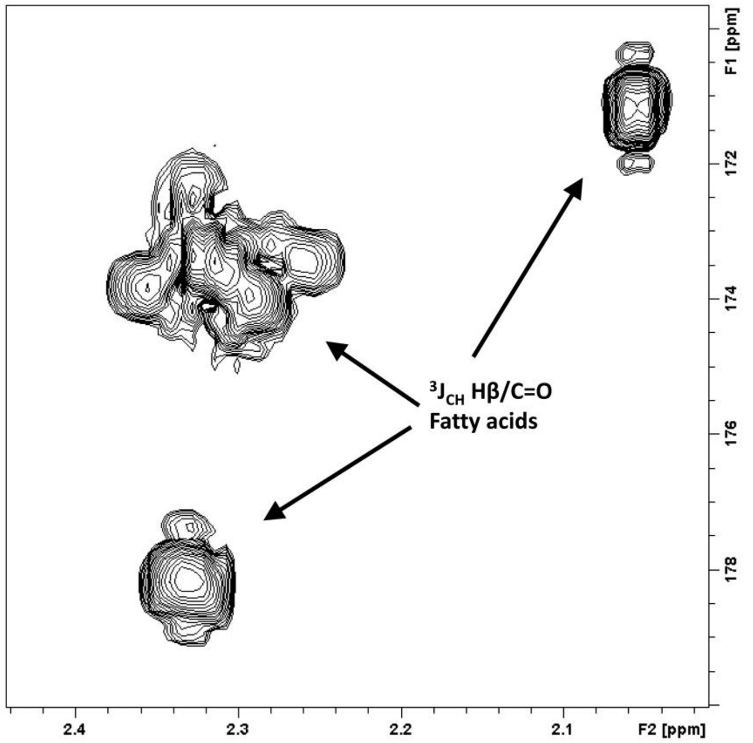

Figure S2. Expanded region of the 2D ^{1}H-^{13}C HMBC spectrum of hexane extract of *N. variegate* showing correlations between fatty acid carbonyls and their adjacent Hβ. The two groups of signals at ~171.6-174 ppm and ~178.2 ppm, respectively, may be accounted for by the presence of esterified and free fatty acids, respectively.

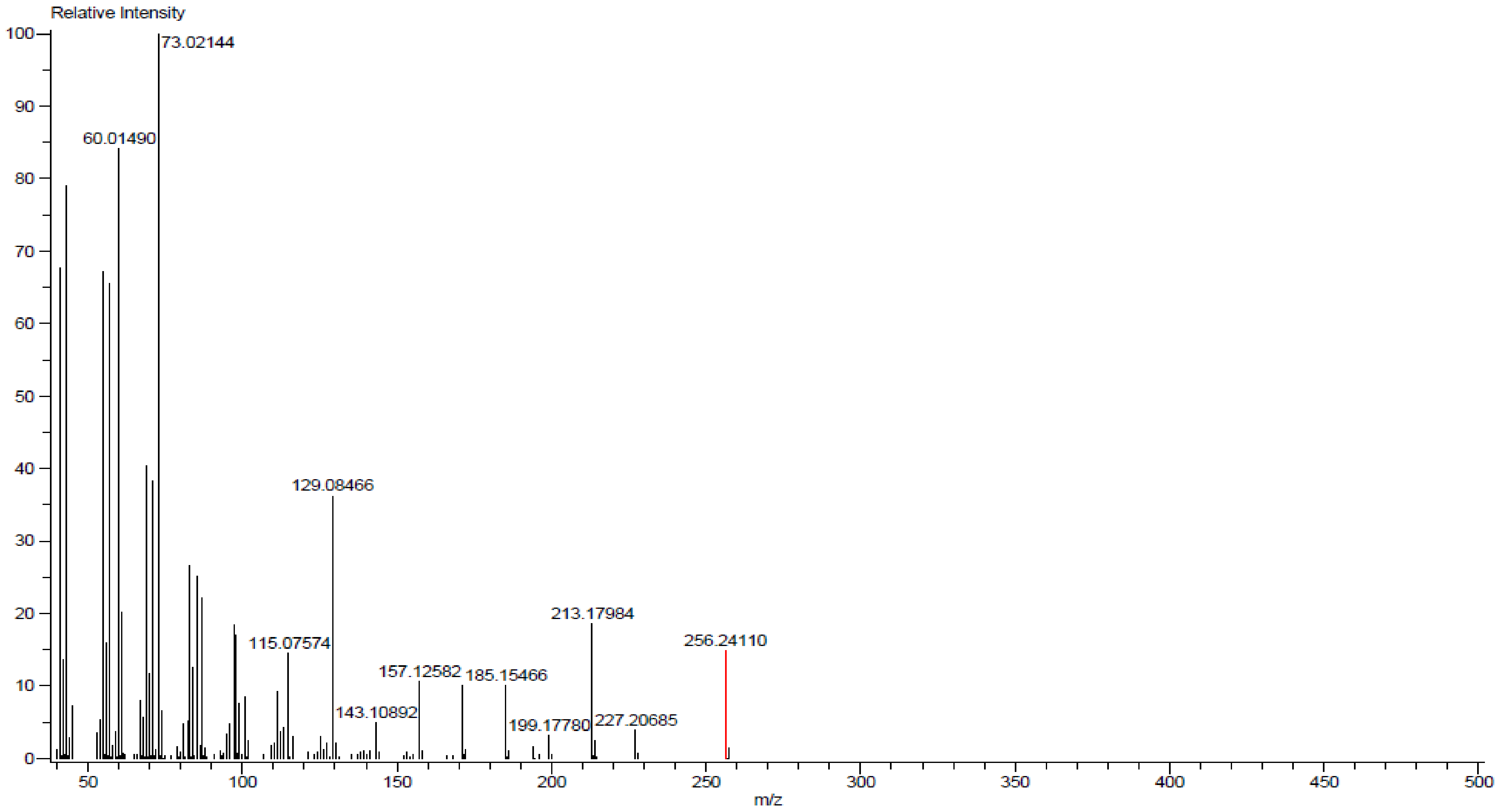

Mass	Intensity	Calc. Mass	Mass Difference (mmu)	Possible Formula	Unsaturation Number
256.24110	8781.95	256.24023	0.87	$^{12}C_{16}{}^{1}H_{32}{}^{16}O_2$	1.0

Figure S3. Mass spectrum of *n*-Hexadecanoic acid (Palmitic acid) (**1**)

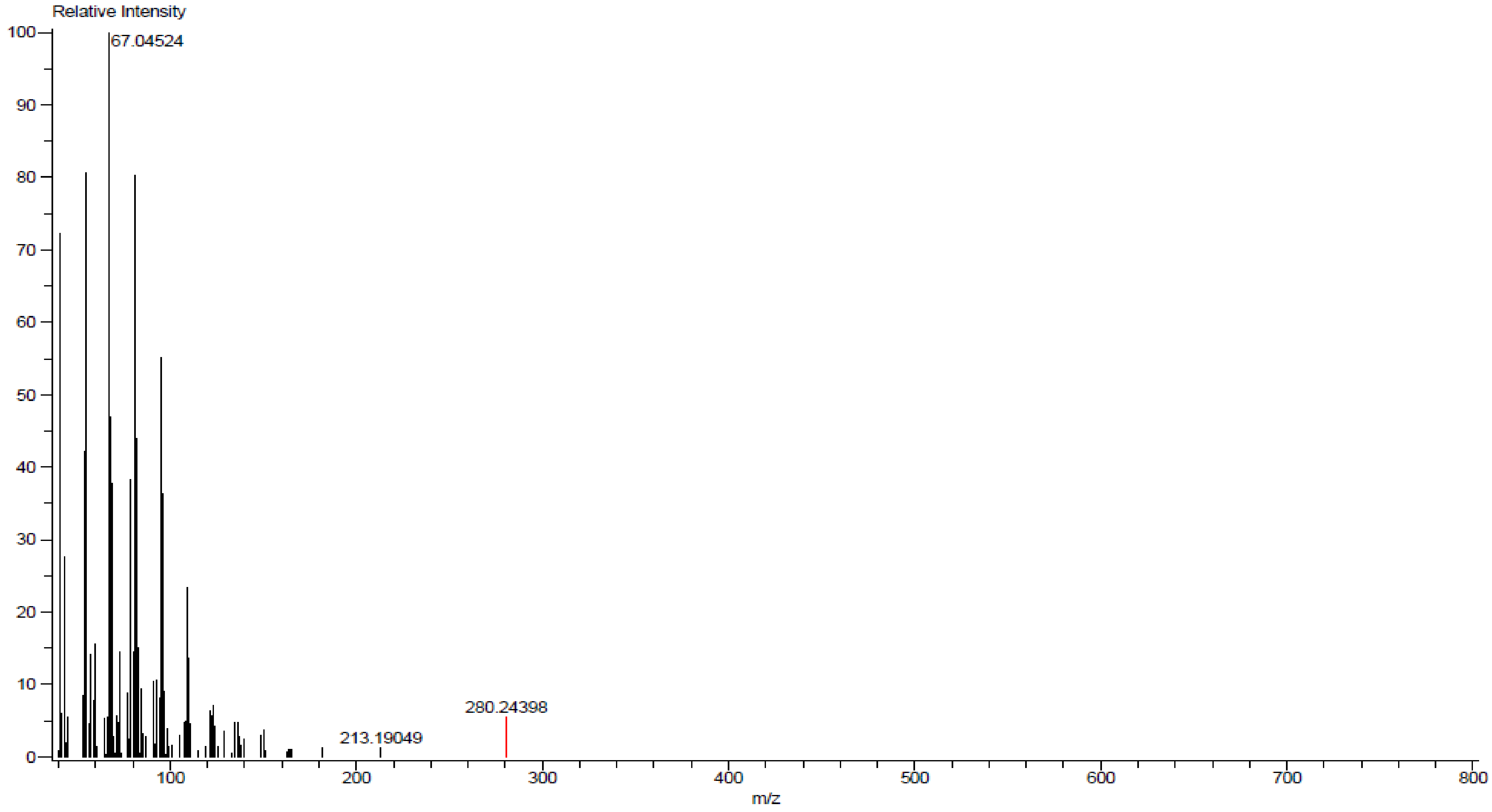

Mass	Intensity	Calc. Mass	Mass Difference (mmu)	Possible Formula	^{12}C	^{1}H	^{16}O	Unsaturation Number
280.24398	1027.48	280.24023	3.75	$^{12}C_{18}{}^{1}H_{32}{}^{16}O_2$	18	32	2	3.0

Figure S4. Mass spectrum of Octadecan-(9,12)-dienoic acid (**2**)

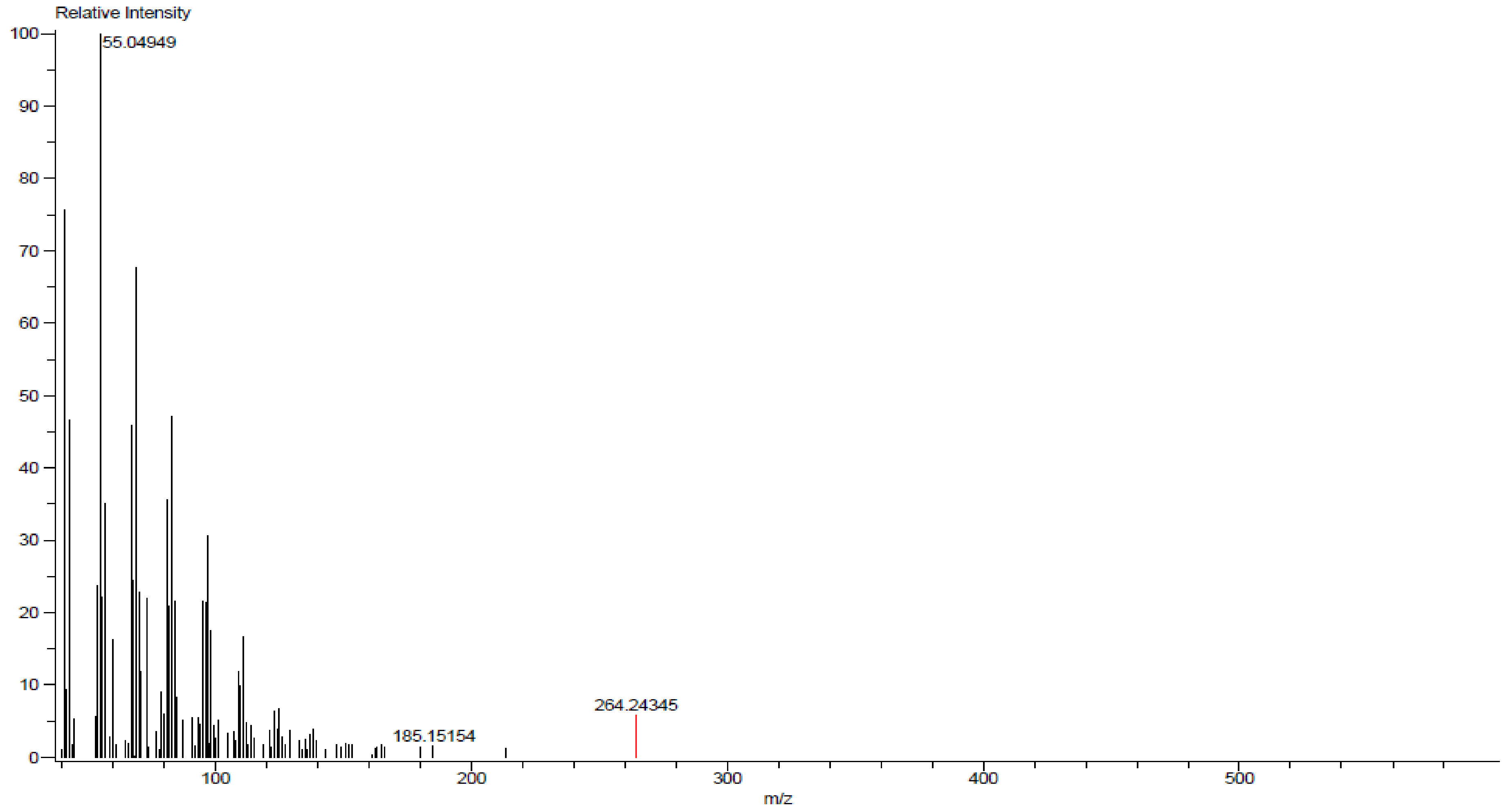

Mass	Intensity	Calc. Mass	Mass Difference (mmu)	Possible Formula	Unsaturation Number
264.24345	1240.14	264.24531	-1.86	$^{12}C_{18}{}^{1}H_{32}{}^{16}O_{1}$	3.0

Figure S5. Mass spectrum of (9*Z*)-Octadec-9-enoic acid (Oleic acid) (**3**)

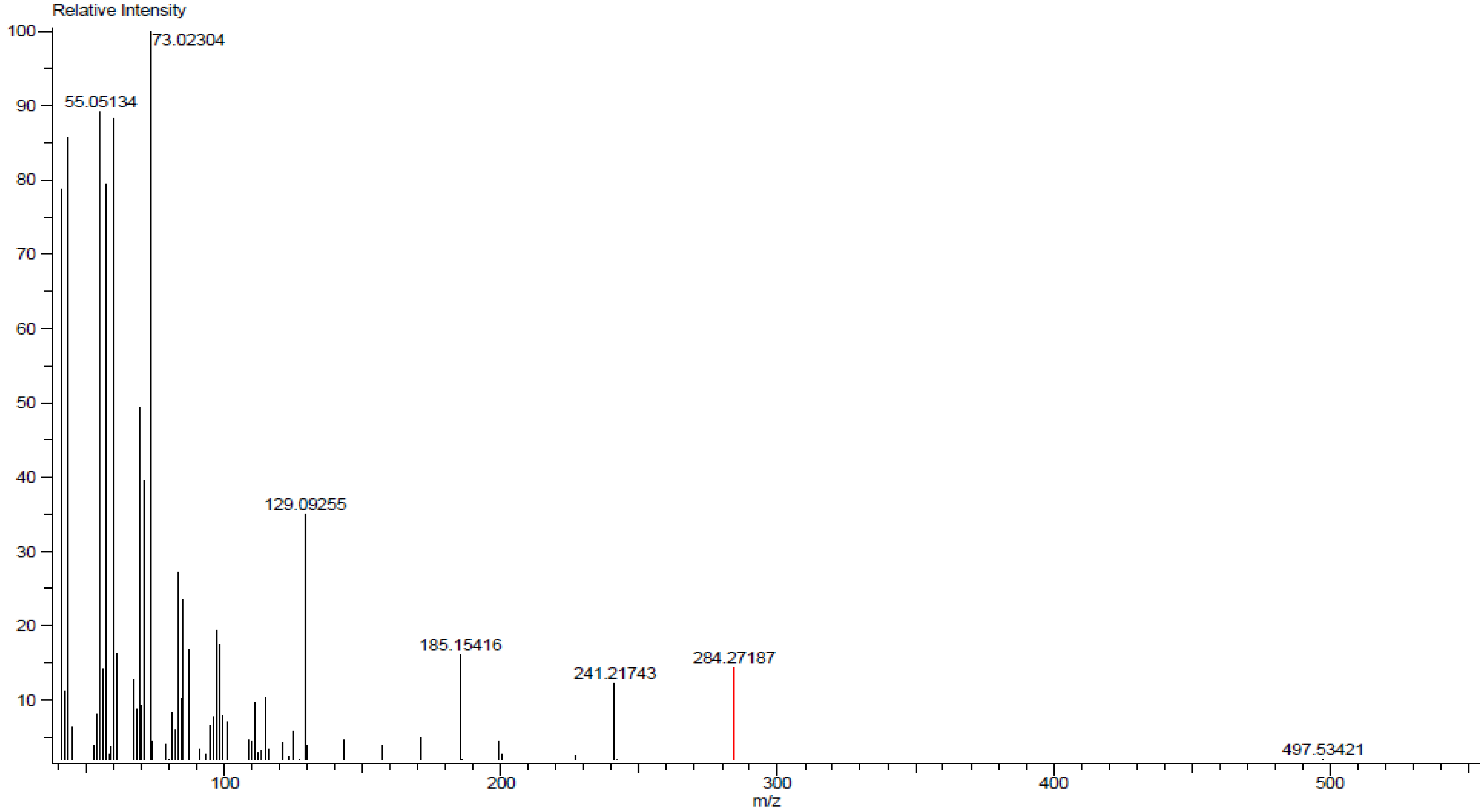

Mass	Intensity	Calc. Mass	Mass Difference (mmu)	Possible Formula	Unsaturation Number
284.27187	1780.55	284.27153	0.34	$^{12}C_{18}{}^{1}H_{36}{}^{16}O_2$	1.0

Figure S6. Mass spectrum of Octadecanoic acid (Stearic acid) (**4**)

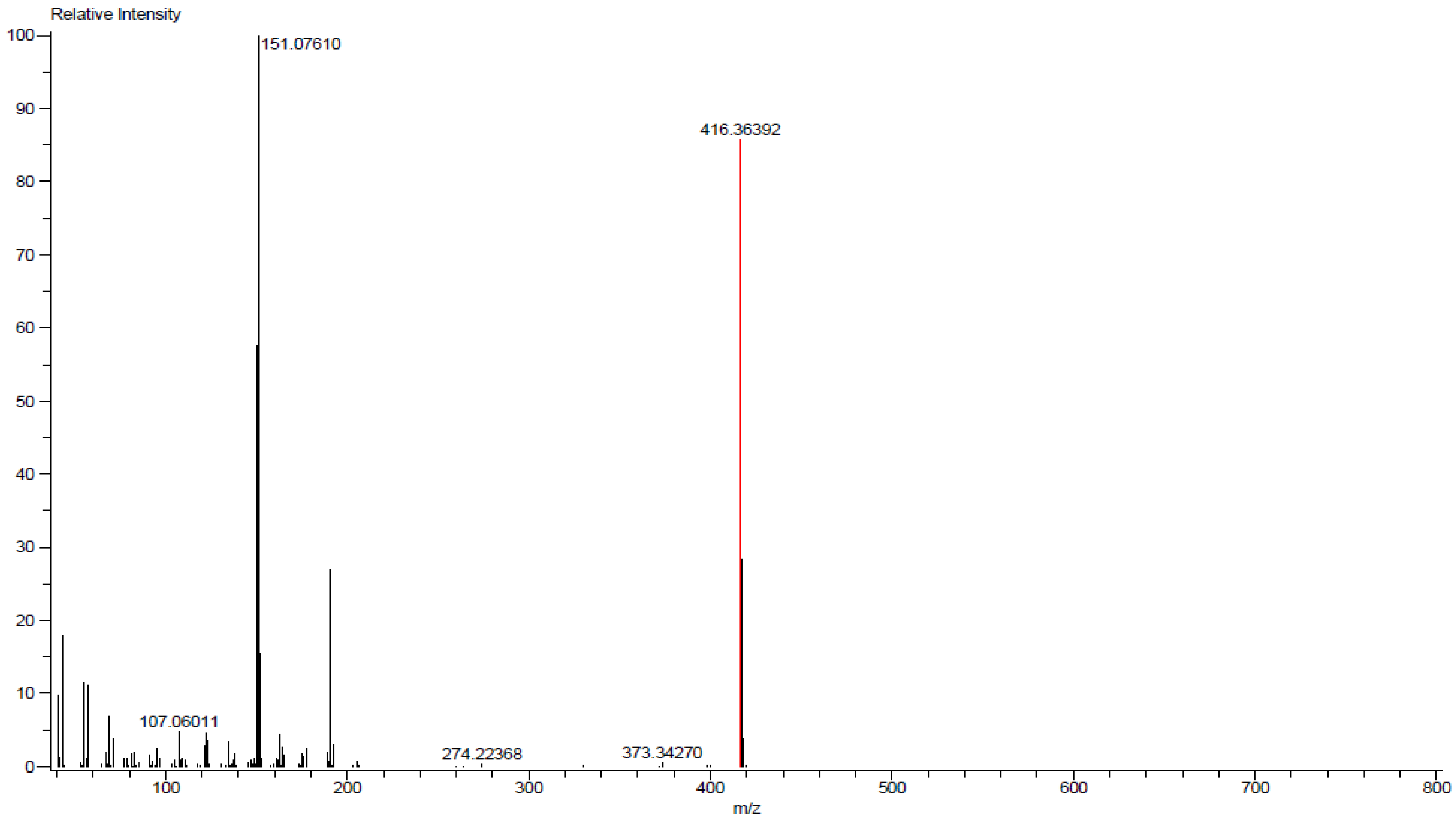

Mass	Intensity	Calc. Mass	Mass Difference (mmu)	Possible Formula	Unsaturation Number
416.36392	66051.91	416.36543	-1.51	$^{12}C_{28}{}^{1}H_{48}{}^{16}O_2$	5.0

Figure S7. Mass spectrum of β-Tocopherol (**11**)

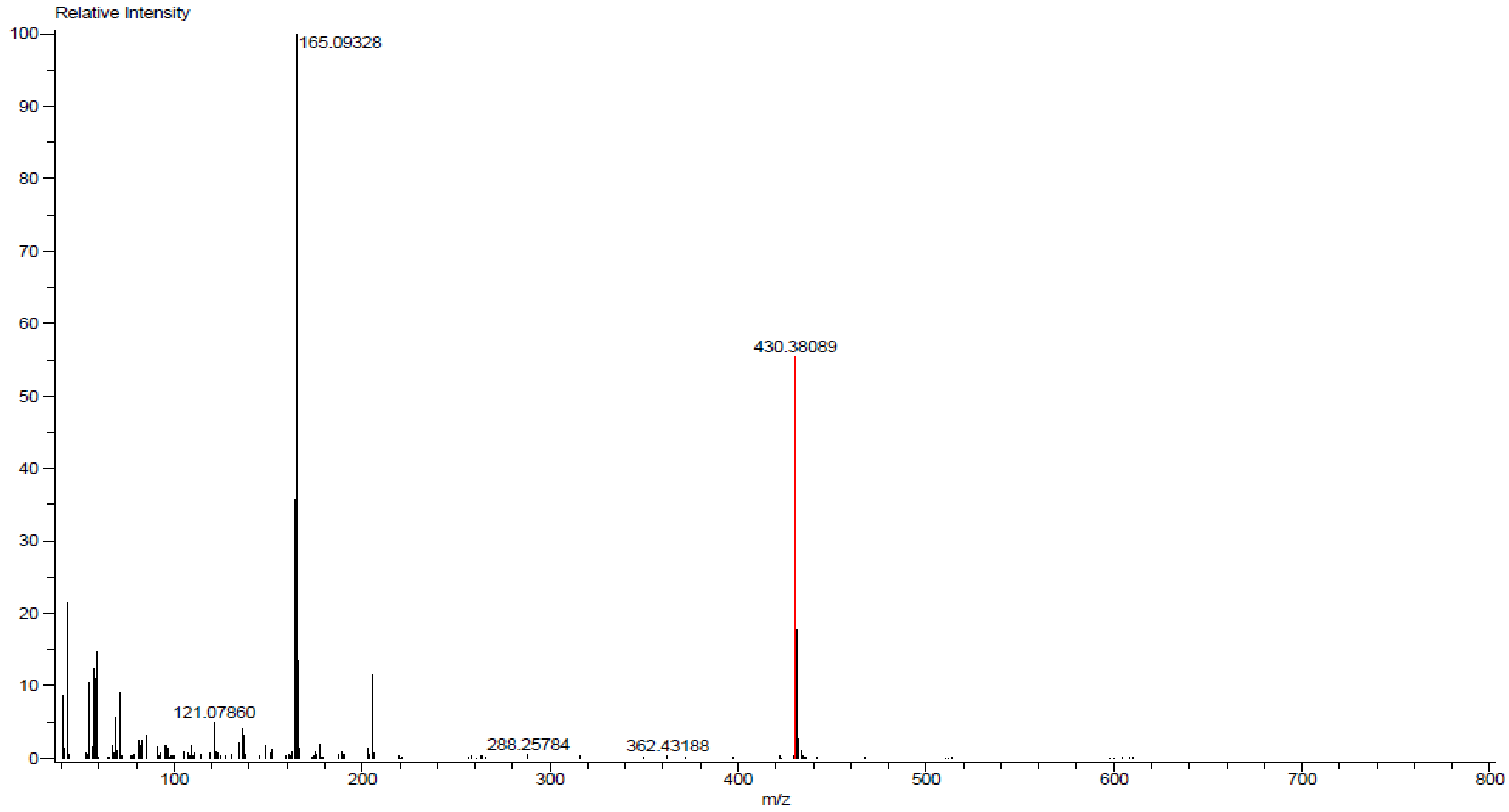

Mass	Intensity	Calc. Mass	Mass Difference (mmu)	Possible Formula	Unsaturation Number
430.38089	52136.45	430.38108	-0.19	$^{12}C_{29}{}^{1}H_{50}{}^{16}O_2$	5.0

Figure S8. Mass spectrum of α-Tocopherol (Vitamin E) (**12**)

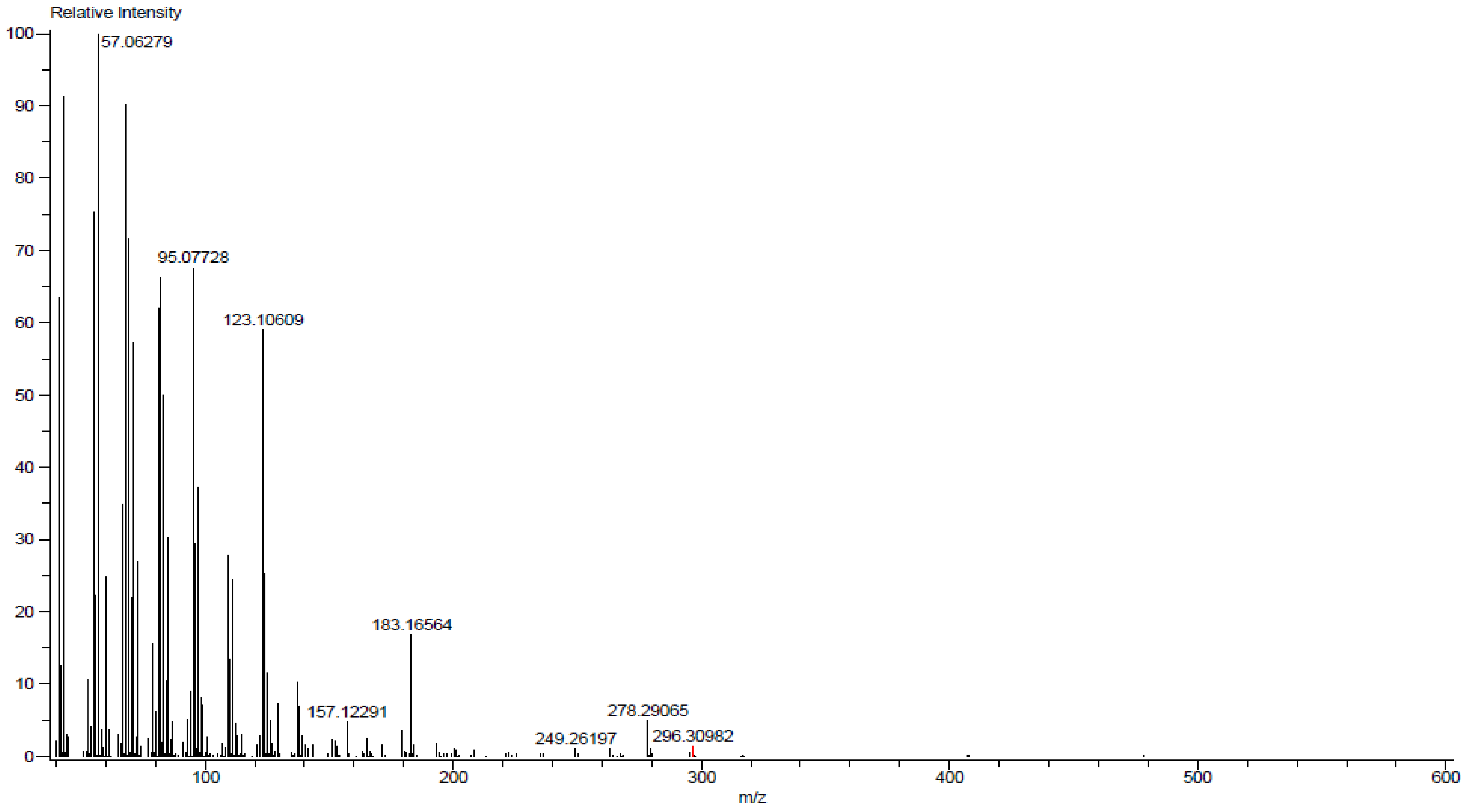

Mass	Intensity	Calc. Mass	Mass Difference (mmu)	Possible Formula	Unsaturation Number
296.30982	1582.69	296.30791	1.90	$^{12}C_{20}{}^{1}H_{40}{}^{16}O_{1}$	1.0

Figure S9. Mass spectrum of (2*E*,7*R*,11*R*)-3,7,11,15-Tetramethyl-2-hexadecen-1-ol (Phytol) (**13**)

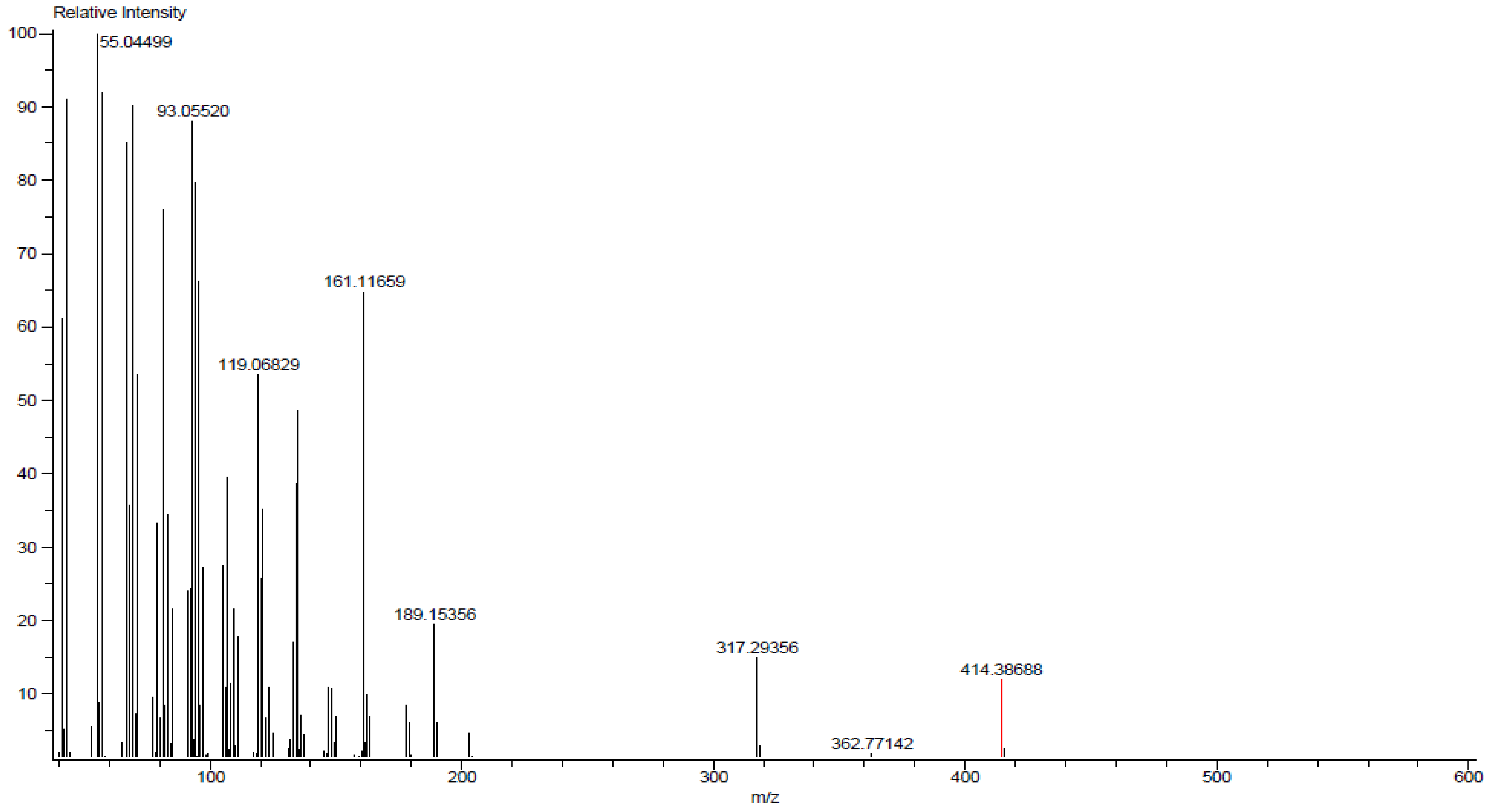

Mass	Intensity	Calc. Mass	Mass Difference (mmu)	Possible Formula	^{12}C	^{1}H	^{16}O	Unsaturation Number
414.38688	2122.34	414.38616	0.72	$^{12}C_{29}{}^{1}H_{50}{}^{16}O_{1}$	29	50	1	5.0

Figure S10. Mass spectrum of Stigmastan-3-one (**14**)

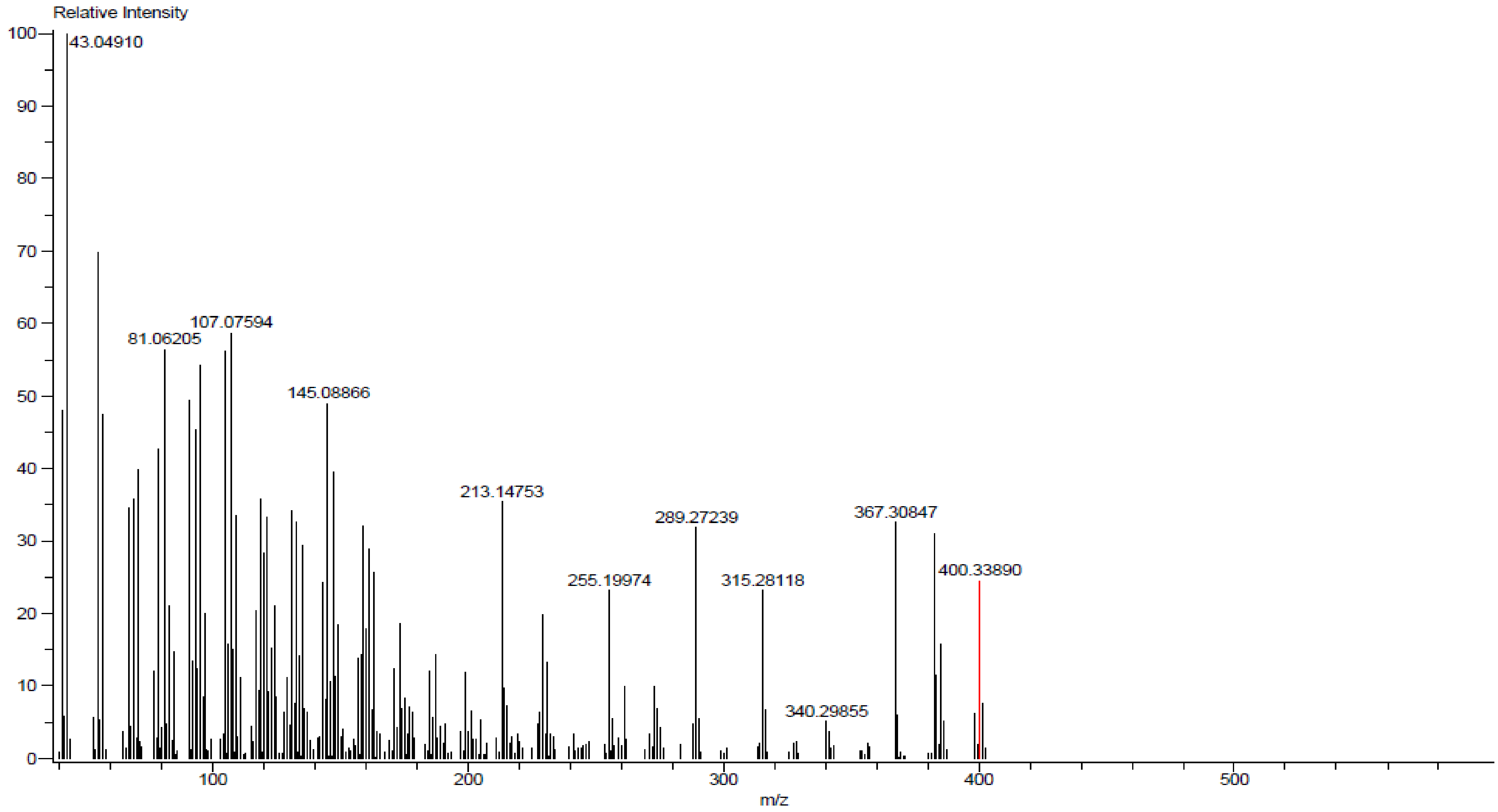

Mass	Intensity	Calc. Mass	Mass Difference (mmu)	Possible Formula	Unsaturation Number
400.33890	8900.78	400.33413	4.77	$^{12}C_{27}{}^{1}H_{44}{}^{16}O_2$	6.0

Figure S11. Mass spectrum of (3β, 24R) Ergost-5-en-3-ol (Campesterol) (**15**)

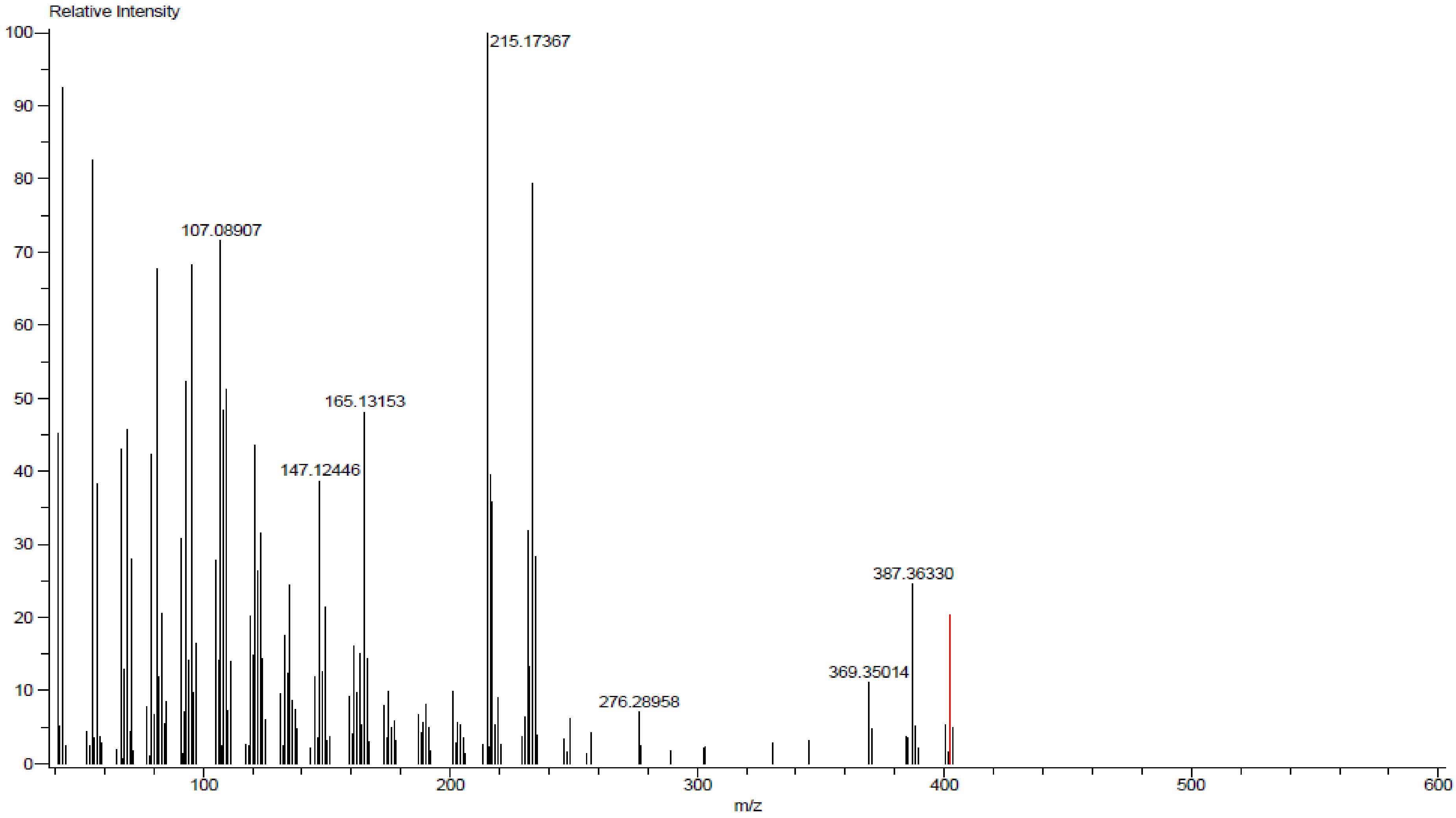

Mass	Intensity	Calc. Mass	Mass Difference (mmu)	Possible Formula	Unsaturation Number
402.38723	3503.88	402.38616	1.07	$^{12}C_{28}{}^{1}H_{50}{}^{16}O_{1}$	4.0

Figure S12. Mass spectrum of Ergostanol (**16**)

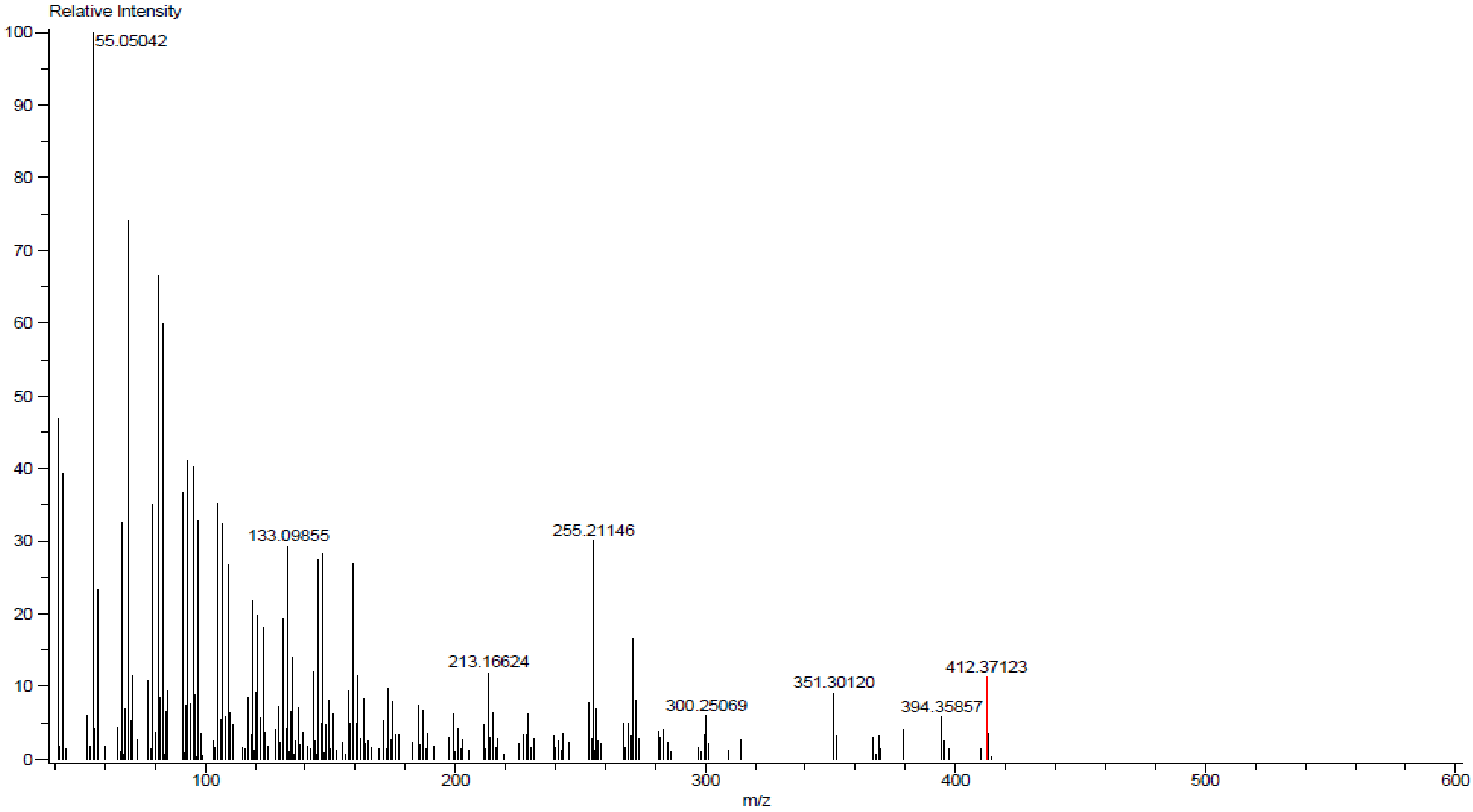

Mass	Intensity	Calc. Mass	Mass Difference (mmu)	Possible Formula	Unsaturation Number
412.37123	2734.74	412.37051	0.71	$^{12}C_{29}{}^{1}H_{48}{}^{16}O_{1}$	6.0

Figure S13. Mass spectrum of Stigmasta-4,22-dien-3-β-ol (**17**)

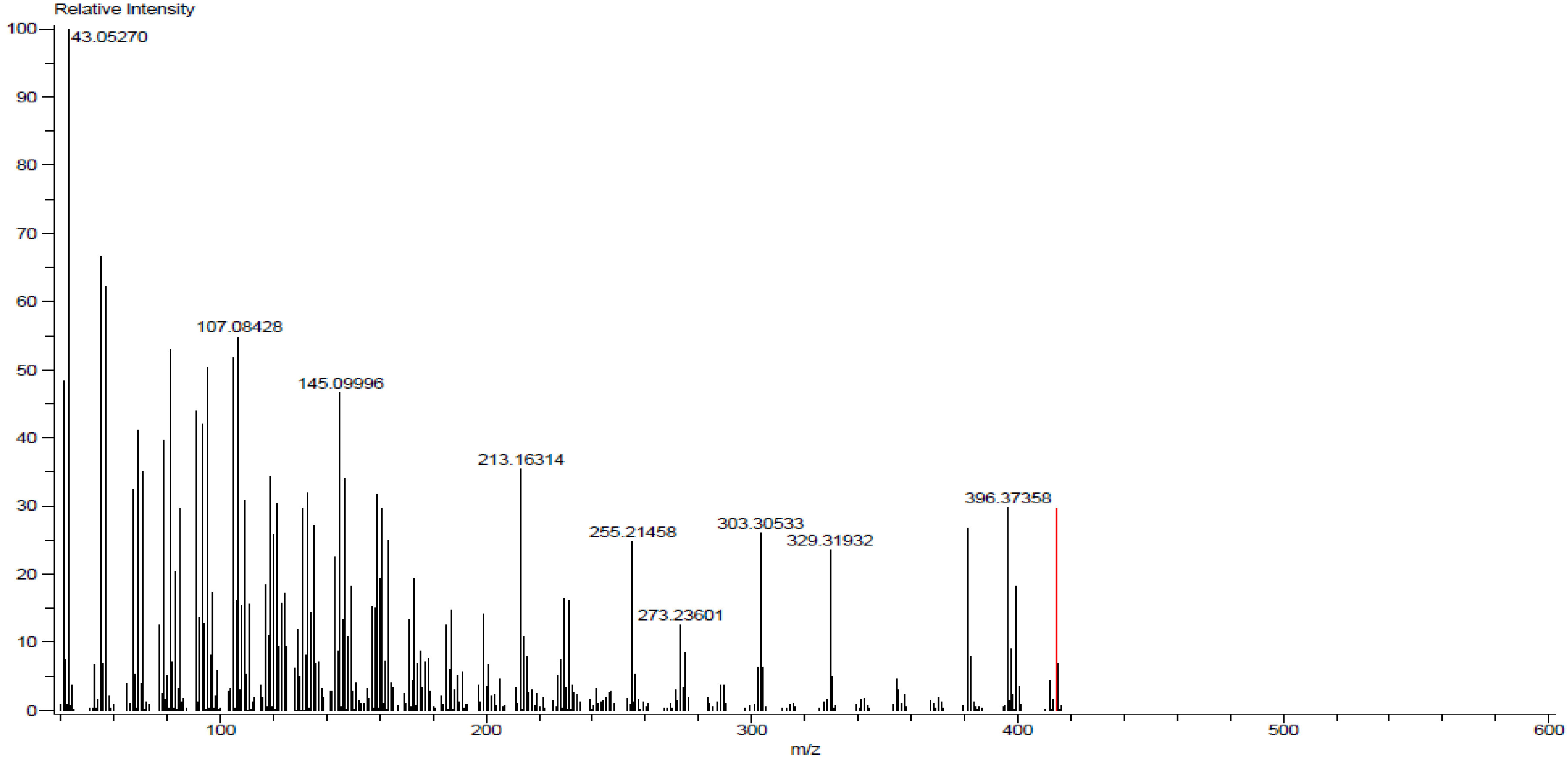

Mass	Intensity	Calc. Mass	Mass Difference (mmu)	Possible Formula	Unsaturation Number
414.38655	22998.02	414.38616	0.39	$^{12}C_{29}{}^{1}H_{50}{}^{16}O_{1}$	5.0

Figure S14. Mass spectrum of β-Sitosterol (**18**)

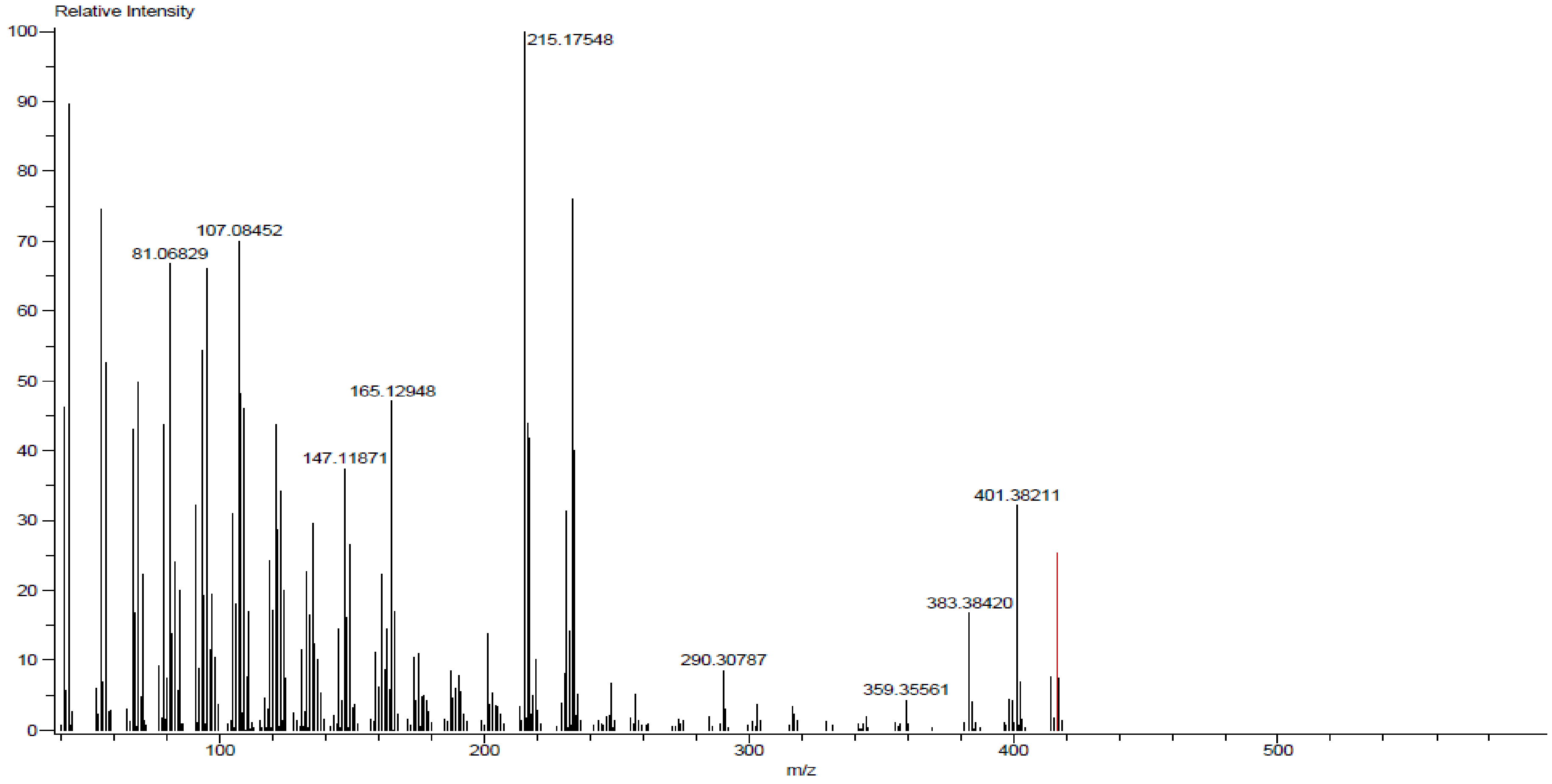

Mass	Intensity	Calc. Mass	Mass Difference (mmu)	Possible Formula	Unsaturation Number
416.40276	13472.06	416.40181	0.95	$^{12}C_{29}{}^{1}H_{52}{}^{16}O_{1}$	4.0

Figure S15. Mass spectrum of Stigmastanol (**19**)

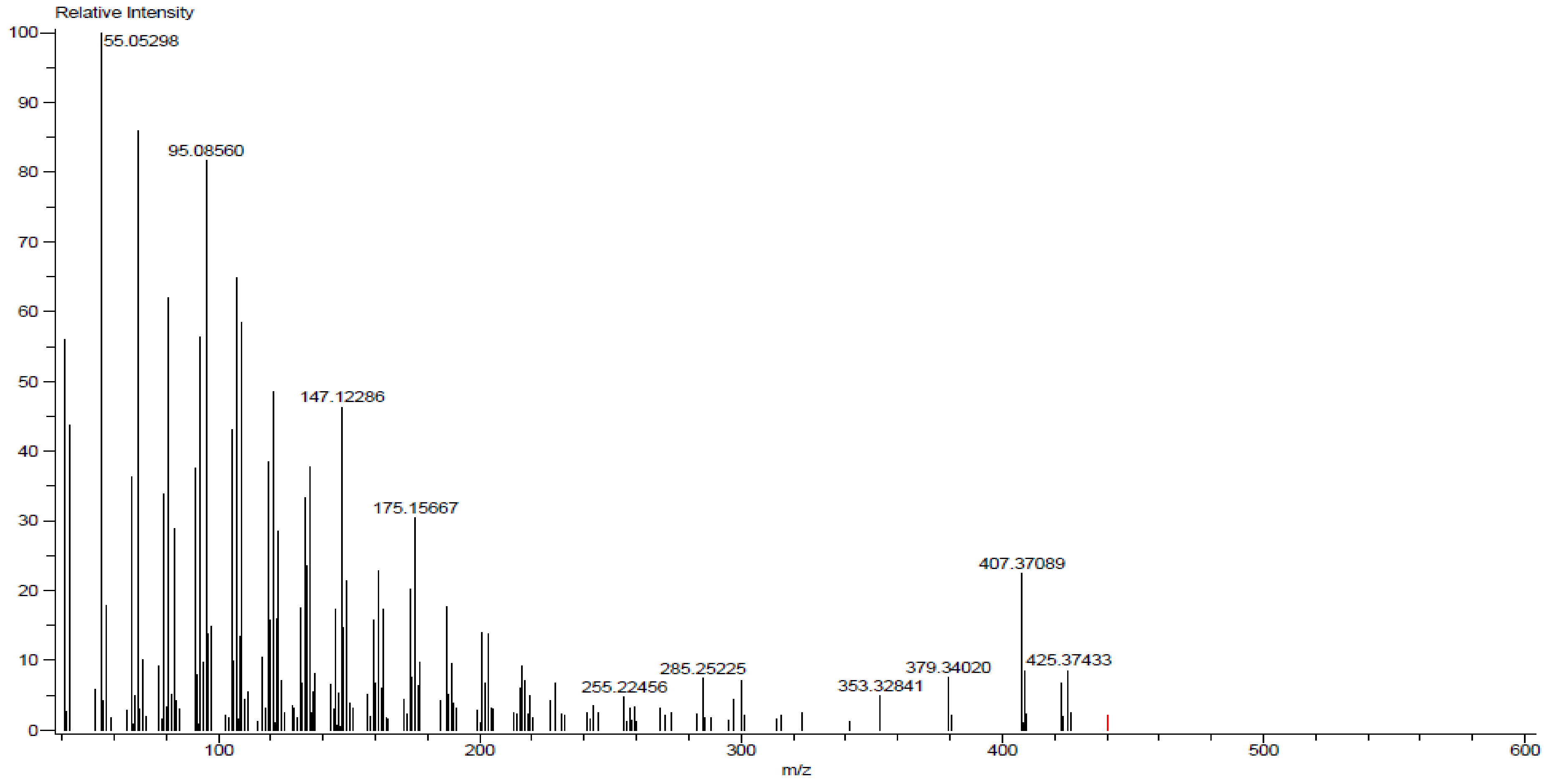

Mass	Intensity	Calc. Mass	Mass Difference (mmu)	Possible Formula	Unsaturation Number
440.40234	333.96	440.40181	0.53	$^{12}C_{31}{}^{1}H_{52}{}^{16}O_1$	6.0

Figure S16. Mass spectrum of 24-Methyl-9,19-cyclolanost-24-en-3-ol-3-β (**20**)
